# Single Super Phosphate Improves *Lolium perenne* Quality and Rhizosphere Microorganism Structure Under Combined Cadmium and Arsenic Stress

**DOI:** 10.3390/toxics13090805

**Published:** 2025-09-22

**Authors:** Toe Toe Maw, Jiangdi Deng, Bo Li, Yanqun Zu, Zuran Li

**Affiliations:** 1 College of Animal Science and Technology, Yunnan Agricultural University, Kunming 650201, China; toetoemaw@yau.edu.mm (T.T.M.); dengjd.cn@gmail.com (J.D.); 2 College of Environmental and Chemical Engineering, Chongqing Three Gorges University, Chongqing 404100, China; 3 College of Resources and Environment, Yunnan Agricultural University, Kunming 650201, China; libo@ynau.edu.cn; 4 College of Landscape and Horticulture, Yunnan Agricultural University, Kunming 650201, China

**Keywords:** phosphorus fertilizer, antioxidant enzyme activities, Cd and As combined contamination, microbial community diversity in rhizosphere soil

## Abstract

Cadmium and arsenic co-contamination found in mining actions indicates major effluence in adjacent farmland soils, disturbing the plant physiology and soil’s microbial community. Phosphorus (P) plays a vital role in reducing soil contamination from Cd and As bioavailability and uptake by plants. However, the right P sources for remediation approaches are critical and still require further research in Cd- and As-contaminated soil. This study aimed to explore the effects of different phosphorus fertilizer sources on *Lolium perenne* growth and its physiological and rhizosphere microbial diversity under combined contamination with Cd and As. Pot experiments were performed with seven treatments including SSP (single super phosphate), DAP (diammonium phosphate), MAP (monoammonium phosphate), CaP (calcium phosphate), HighCaP (high calcium phosphate), RP (rock phosphate), and no phosphorus fertilizer application (CK) with five replications in the RCB design. The SSP treatment showed the greatest plant height (15.7 cm), hay yield (3567.6 kg·ha^−1^), and enhanced antioxidant defense activities. It also achieved the highest phosphorus accumulation rate (0.63 g·kg^−1^) with reduced Cd and As uptake. In addition, SSP promoted higher non-protein sulfhydryl (NPT) and phytochelatin synthetase (PCs) contents along with γ-glutamylcysteine synthetase (γ-ECS) activity, and enriched the rhizosphere microbial community, where the Sphingomonas abundance was 7.08% higher than for other treatments. Therefore, this result indicates that SSP can improve the yield and physiology in *L. perenne*, as well as soil the rhizosphere microbial community structure, while reducing Cd and As accumulation in plants under Cd and As stress.

## 1. Introduction

Co-contamination by arsenic (As) and cadmium (Cd) arises from various pollutants associated with household, agricultural, and industrial processes, such as those from garbage incineration [1]. Although most mining activities for minerals have ceased, the solid waste, sewage, and waste gases generated during mining, washing, and transportation continue to introduce significant quantities of metalloids and heavy metals in the ecosystem [2,3]. In some regions, the levels of these contaminants greatly exceed the normal background levels in the soil, potentially leading to harmful environmental impacts [4]. One of the primary ecological issues remains the remedy and management of heavy metal-polluted soils surrounding mining regions [5], driving interest in soil remediation studies [6]. Numerous studies have shown considerable Cd deposition in regions impacted by mining in southern China, especially in tin and lead–zinc mines. Concentrations of Cd were markedly increased in the soil and rice grains within a tin mining region [7]. Furthermore, it has been estimated that heavy metal contamination affects approximately 20% of China’s cropland, and human activities are expected to exacerbate this condition over the coming decades, particularly in land used for agriculture [8]. A buildup of heavy metallic elements in soil would result in several issues, including decreased agricultural productivity, soil quality, and microbial biodiversity, ultimately endangering the health of humans and animals through food supply [9,10].

Among metals in contaminated soil, Cd is the most mobile element, which can build up in crops in significant quantities without causing phytotoxicity. Both human health and natural ecosystems may be severely and frequently irreversibly impacted by the hazardous pollutant Cd [11]. Typically related to metals including Cd, Cu, Pb, and Zn, As is another element that can be found in greater concentrations at mine sites [12]. These contaminants exacerbate ecological issues by negatively affecting plants in croplands and contaminating nearby waterways and the air [13]. According to numerous investigations, plants are adversely affected by large amounts of Cd in the soil, resulting in stunted development and necrosis of the leaves [14], disruption of minerals and the metabolism of carbohydrates [15], and reactive oxygen species (ROS) generation, which significantly lowers production [16]. As a consequence of numerous metabolic processes in distinct cell division, ROS are often continually generated in plants [17]. Nevertheless, after being exposed to stress from heavy metals, ROS production may exceed the antioxidant’s ability to scavenge, resulting in oxidative stress and harm to biological molecules [18]. Plants have antioxidant protective mechanisms that include decreases in glutathione (GSH), nonenzymatic antioxidants, and enzymes of antioxidants such as catalase (CAT), peroxidase (POD), and super-oxide dismutase (SOD), which regulate ROS production and defend the compartments [19]. The SOD–CAT–POD mechanism detoxifies the ROS formed from heavy metals by converting superoxide anions toward H_2_O_2_ and O_2_, such as CAT, as well as various peroxidases, with oxidation complementing the recovery process [20].

In reality, soil microbes and micro-ecosystems are affected by toxic metal pollution [21]. The architecture of rhizosphere microbial communities and their functioning metabolic processes can be changed by exposure to toxic metals [22,23]. The presence of Cd reduces the diversity of microbial groups and the processes of soil enzymes by altering the biological function and community structure of soil microorganisms [24,25]. Soil microbes are essential parts of soil environments and significantly influence plant development [26]. They help with nutrient cycling and enhance stress resistance, which includes bacteria such as Proteobacteria, Actinobacteria, and Bacteroidetes. As adsorption, oxidization and reduction, leakage, modification, and volatilization are only a few of the processes by which microbiology might clean up arsenic-contaminated sites [27]. Microorganisms are primary factors that facilitate phytoremediation owing to the multipart interactions between crops and microbes in the rhizosphere [28,29]. As a result of all of the relationships and alteration processes present in co-contaminated soil, As and Cd remediation is more complicated than the remediation of specific metals [30]. Biological, physical, and chemical approaches are three primary remediation techniques for soils contaminated with heavy metals [31]. Chemical immobilization and phytoremediation methods have been commonly utilized recently to remediate As- and Cd-contaminated soil for the elimination of heavy metals with phytoextraction, soil washing, or electrodialysis [32,33] or conversion of these metals into less hazardous forms through ion adsorption, the precipitation of chemicals, and then the phytostabilization process [6].

The Gramineae family includes the ryegrass variety *Lolium perenne*. Apart from its rapid growth rate, flexibility, quick development, and enormous biomass, *L. perenne* also exhibits excellent resistance and tolerance to toxic chemicals [34]. Certain physicochemical variables, such as shoot growth, fresh weight, soluble protein and chlorophyll contents, antioxidative enzymes, and numerous relation gene functions involved in cellular metabolism or Cd transportation, are impacted under Cd stress, even though ryegrass can withstand relatively high Cd contents [35]. Demonstrating the hostile influences of Cd on cellular metabolism and the physiological response of ryegrass to the presence of Cd may also shed light on the processes underlying ryegrass’s capacity to withstand and retain elevated Cd concentrations. In addition to ryegrass’s physiological reaction to Cd, the microbes that inhabit it might also be crucial to how it reacts to Cd stress–immobilization in situ. According to earlier studies, ryegrass (*L. perenne*) may be a suitable species for bioremediation of Cd-polluted soils, as it can absorb Cd through its aboveground parts and roots [36]. A popular technique for cleaning heavy metal-contaminated soil in the field is in situ immobility, which is less damaging, less expensive, and poses little environmental danger [37].

According to Lin et al. [38], chemical immobilization also entails adding remedies to polluted soil, which changes the chemical properties or types of Cd and As. These techniques provide numerous benefits, including clarity, financial viability, sustainability, and efficiency, while causing minimal disturbance to the soil [39]. One essential supplemental phase for the remediation of soil polluted with toxic metals is phosphorus fertilizer application [40]. One of the three main macronutrients needed for plant growth, phosphorus is essential for transferring energy, photosynthesis, and the production of proteins and nucleic acids, among other metabolic functions [41]. Additionally, phosphorus reduces the harmful effects of Cd by influencing how it functions in the soil [42]. Due to the various rates of release and plant access, SSP and DAP are the most commonly employed of these fertilizers [43].

Effective approaches for handling Cd–As co-contaminated soil will be made possible by understanding the exact relationships between Cd and As in plants, as well as remedial techniques [44]. Finding the best P resource is necessary to improve the effectiveness of fertilizer usage, while the P recovery efficiency is poor. Furthermore, it is essential to determine how various P sources impact plant growth and the antioxidant defense system [45]. Therefore, this study aimed to examine the impacts of different phosphorus fertilizer applications on the physiological and rhizosphere soil bacterial responses of *L. perenne* to these P sources, providing an alleviating effect on Cd and As contamination in soil. The hypotheses were that (1) among the different phosphorus fertilizer applications, an appropriate P source would help mitigate Cd and As contamination in *L. perenne*, and (2) phosphorus application would influence the physiological responses and soil microbial diversity under Cd and As stress, thereby providing an effective strategy for managing contaminated soils and ensuring forage crop quality.

## 2. Materials and Methods

### 2.1. Materials and Experimental Design

#### 2.1.1. Materials

Soil samples were especially polluted with Cd and As and collected from a mining area in Lanping city (26.459702″ N, 99.474214″ E) with an elevation of 2345 m (7694 ft), Yunnan province, China. Soil samples were collected and air-dried in the shed. A 2 mm mesh sieve was then used to remove unnecessary materials, such as stones, plant roots, and debris, in preparation for planting. Some chemical analyses were conducted by storing the soil samples at 4 °C. The basic soil data are as follows: pH 8.75, total organic carbon 5.23 g·kg^−1^, organic matter content 9.01 g·kg^−1^, available phosphorus (AP) 6.26 mg·kg^−1^, total cadmium (Cd) 19.64 mg·kg^−1^, and total arsenic (As) 33.58 mg·kg^−1^. The test plant was a perennial ryegrass, Venus (*Lolium perenne*).

The phosphorus fertilizers included single superphosphate, diammonium phosphate, monoammonium phosphate, calcium phosphate, high calcium phosphate, and rock phosphate. The available Cd and As concentrations from different phosphorus fertilizers are shown in Table 1.

#### 2.1.2. Experimental Design

The study area was situated in Yunnan at coordinates of 25°14′30″ N, 102°05′627″ E, with an elevation of approximately 1819 m, with a subtropical monsoon climate, characterized by an annual average temperature of 14.9 °C and average yearly rainfall of approximately 1000.5 mm. Most of the rainfall occurs between May and September.

There were seven treatments, each using a different phosphorus fertilizer application, under Cd and As co-contaminated soil, with five replications in the RCB design. Different sources of phosphorus fertilizer were used at an equal rate of 56 P_2_O_5_ kg·ha^−1^ and applied as basal applications before transplanting. The following treatments were included: P1 = CK (no phosphorus fertilizer application), P2 = SSP, P3 = DAP, P4 = MAP, P5 = CaP, P6 = HighCaP, and P7 = RP.

### 2.2. Research Methods

The soil was thoroughly mixed with urea (500 mg) and K_2_SO_4_ (500 mg) and then applied as a basal fertilizer to each pot, using an appropriate amount of soil at a time. The sample were then placed into plastic pots. Next, 3 kg of soil per pot was prepared and weighed to grow ryegrass seedlings, with 20 plants per pot. Seeds of *L. perenne* were sown in the greenhouse soil. After a month, the plants were ready for transplantation, and the seedlings were moved to experimental pots. Different sources of phosphorus fertilizer were applied as basal applications before transplanting. Water was used to adjust the maximum moisture content to about 60% of the water-holding capacity of the soil.

Plants were measured by their height in centimeters (cm) above the base of the plant at the soil’s surface to the plant’s tallest height in weekly intervals. Sixty days after transplanting, the ryegrass plants were collected and weighed to determine their fresh weight. The collected plants were allocated into shoots and roots. Then, the roots were cleansed with tap water and scanned. Scanned images were captured using an scanner (Perfection V800, Epson, Indonesia) and analyzed the root morphology using WinRHIZO Pro software. Some harvested plants were cleaned with tap water and kept at −85 °C for 30 min to prepare fresh samples for the analysis of antioxidant enzyme activities, including SOD, POD, CAT, MDA, NPT, GSH, PCs, and γ-ECS measurements. Then, some plants were placed in an oven at 70 °C for 48 h to achieve a stable weight and then ground and placed into a 40-mesh screen to test the amounts of Cd and As and total P concentration in the plants.

After the plants were removed, some soil samples were also immediately taken using a box with nitrogen liquid for the determination of the rhizosphere microbial community and diversity. Then, the sample soils were collected, air-dried, powdered, and cleaned using a 2 mm sieve and stored for later use.

### 2.3. Laboratory Methods

The POD activity in plants was measured using a 4-methyl catechol substrate. Spectrophotometry was employed to detect the H_2_O_2_-induced oxidation at an absorbance rate of 420 nm. At ambient temperature, the reacted liquid consisted of 3.0 mL, containing 5 mM of 4-methyl catechol, 5 mM of H_2_O_2_, 500 µL of raw extracts, and 100 mM of Na_3_PO_4_ buffer (pH 7.0). Each function in these testing procedures was described as a 0.001 absorbance difference per minute [46].

The plant’s SOD activity was assessed using the SOD enzyme to inhibit the photoreduction of nitroblue tetrazolium (NBT) [47]. Next, 0.1 mM pf EDTA, 50 mM of buffered sodium phosphate (pH 7.6), 50 mM of carbonate of sodium, 50 µM of NBT, 12 mM of L-methionine, 10 µM of riboflavin, and 100 µL of the extracted natural product were blended to create the solution for the reaction, which had a final volume of 3 mL. After 15 min at ambient temperature, the mixture was exposed to white light to start the SOD reaction. Following 15 min of incubation, a spectrophotometer set at 560 nm was used to measure the SOD activity. At 50% suppression of the photochemical loss of NBT, the enzyme activity was measured.

The CAT activity in the plants was measured by assessing the absorbance at 240 nm using a spectrophotometer at room temperature. This measurement reflects the breakdown of H_2_O_2_ [48]. In the assay, the CAT activity is characterized by a change in absorbance of 0.001 per minute. The reaction mixture was prepared to a total volume of 3 mL by combining 100 µL of crude extract, 30 mM of H_2_O_2_, and 100 mM of sodium phosphate buffer (pH 7.0).

MDA content in plant: 0.1 g of plant material was placed into 2 mL centrifuged tubes, and a 5% trichloroacetic acid mixture was added to determine the MDA value. After centrifuging the contents for 25 min at 14,000× *g*, 0.5 mL of the solution was transferred into 1 mL of 20% C_2_HCl_3_O_2_ acid, containing 0.5% C_4_H_4_N_2_O_2_S acid. 200 µL of supernatant was extracted and centrifuged at 10,000× *g* for 10 min. Then, the clear liquid was placed in 100 °C bathwater for 30 min. Following measurements of its absorbance at 532 and 600 nm, the MDA concentrations (µmol g^−1^ FW) were computed by deducting the generalized absorbance at 600 nm [49].

**NPT content in plants:** Here, 0.4 mL of PBS (0.1 mol/L) and approximately 0.1 g of plant material were placed in a grinder and homogenized on ice. Following this, methanol was added and stirred for 10 min at 25 °C to prevent the evaporation of the liquid. The supernatant obtained after centrifugation at 4 °C for 10 min at 12,000 rpm was used for NPT content measurements at 412 nm using a spectrophotometer (model V-5800, Shanghai Precision Instrument Co., Ltd., Shanghai, China) [50].

**GSH levels in plants:** The solution was prepared by mixing 1 mL of 5% sulfosalicylic acid with 0.1 g of the sample. An ice bath was prepared to place the mix. Then, it was centrifuged at 12,000 rpm for approximately 10 min at 4 °C. Glutathione (GSH) was measured using a spectrophotometer (model V-5800, Shanghai Precision Instrument Co., Ltd., China) [50].

**PCs content in plants:** By deducting GSH contents in the volumes of all NPTs as PCs (µmol·g^−1^) = NPT − total GSH, the total was determined and reported as µmolg^−1^ [51].

**γ-ECS activity in plants:** Approximately 0.5 g of fresh plant matter was processed using liquid nitrogen to create a powder. This powder was then mixed with 4.5 mL of PBS at a concentration of 0.01 mol L^−1^ (pH = 7.4). The resulting solution was centrifuged at 4500 rpm in 15 min. To measure the γ-ECS activities, γ-ECS test kits from Suzhou Grace Biotechnology Co., Ltd. (Suzhou, China) were used, with measurements taken at 450 nm. The results were reported in µmol/h/g [50].

**P content in plants:** A continuously flowing analysis instrument (AA3, Germany) was used to measure the TP concentrations after the aboveground portions of *L. perenne* were dried for approximately 30 min at 70 °C to a constant weight. Additionally, 0.3 g samples were heated for 30 min in H_2_SO_4_ and H_2_O_2_ (30%) to analyze the total P [52].

**Cd content in plants:** Samples were crushed into powder and placed in an oven at 60 °C. The biomass weight was recorded, and 15 mL of a tri-acid mixture [53] was prepared. This mixture consisted of sulfuric acid (H_2_SO_4_, 18M, 96%), concentrated nitric acid (HNO_3_, 16M, 71%), and perchloric acid (HClO_4_, 11M, 71%) at a ratio of 5:1:2. The plant material (1 g) was digested using this blend at 60 °C for 30 min. An AAnalyst 600 atomic absorption spectrophotometer (PerkinElmer Inc., MA, USA) was used to measure the Cd.

**As content in plants:** A plant sample of about 0.300 g was used, and 5 mL of nitric acid was also placed in a Teflon digestion tube. After 8 h, the hydrogen peroxide (30%) was mixed and kept at 140 °C for 4 h in the oven. Then, it was heated with a hot plate at 150 °C. When the solution reached approximately 1 mL, it was cooled and transferred to a 50 mL volumetric flask, and an atomic fluorescence spectrometer (LC-AFS 9600, Haiguang, Tianjin, China) was used to determine the As concentration [54].

### 2.4. Calculation and Data Analysis

The associations between physiological traits and the contents of Cd and As in *L. perenne* were examined using a Pearson analysis. Statistix (Version 8) was used to handle data on variables under different fertilizer P applications for a one-way ANOVA (LSD, *p* < 0.05). The calculation for hay yield are as follows:

W = N/S × 10,000

where W is the hay yield in kg·hm^−2^, N is the hay yield per pot kg, with the pot area measured in m^2^.

## 3. Results

### 3.1. Effects of Different Phosphorus Fertilizer Applications on Plant Growth, Hay Yield, and Root Morphology of Lolium perenne

#### 3.1.1. Plant Height of *L. perenne*

**Figure 1:**
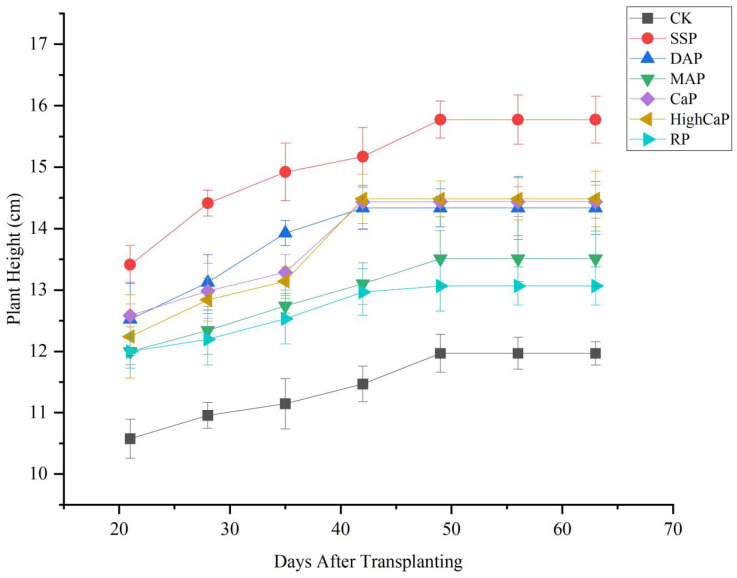
Effects of different phosphorus fertilizer applications on plant height of *Lolium perenne* under Cd- and As-contaminated soil. Notes: CK: no phosphorus fertilizer application, SSP: single super phosphate; DAP: diammonium phosphate; MAP: monoammonium phosphate; CaP: calcium phosphate; HighCaP: high calcium phosphate; RP: rock phosphate.

Compared to all different phosphorus fertilizer applications under Cd- and As-contaminated soil, the plant height showed a significant difference (*p* < 0.05) during the growing period and a trend of increasing first, followed by no further growth in the later period (Figure 1). Higher plant growth was found from 21 to 49 days after transplanting. After 49 days, the plant growth was retarded and we observed linear growth. In all treatments, SSP led to a higher plant height than the other treatments during the first 21 days after growing, followed by CaP, DAP, and HighCaP. The highest plant height was observed in the SSP application treatment at 49 days after transplanting. CK resulted in the lowest plant height among all treatments.

Compared to all different phosphorus fertilizer applications under Cd- and As-contaminated soil, the plant height showed a significant difference (*p* < 0.05) at 35 and during 49–63 days after transplanting and a trend of increasing first, followed by no further growth in the later period (Figure 1). Higher plant growth was found from 21 to 49 days after transplanting. After 49 days, the plant growth was retarded and observed to be linear. In all treatments, SSP had a higher plant height than the other treatments during the first 21 days after growing and was followed by CaP, DAP, and HighCaP. The highest plant height was observed in the SSP application treatment at 49 days after transplanting. CK had the lowest plant height among all treatments.

#### 3.1.2. Biomass Production of *L. perenne*

**Figure 2:**
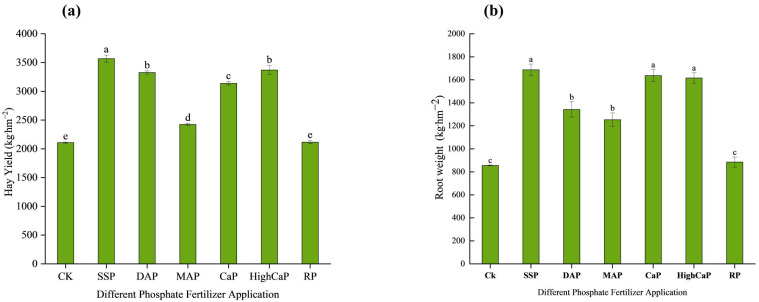
Effects of different phosphorus fertilizer applications on (**a**) shoot biomass and (**b**) root biomass of *Lolium perenne* under Cd- and As-contaminated soil. Notes: CK: no phosphorus fertilizer application; SSP: single super phosphate; DAP: diammonium phosphate; MAP: monoammonium phosphate; CaP: calcium phosphate; HighCaP: high calcium phosphate; RP: rock phosphate. Different letters correspond to significant differences among different treatments (*p* < 0.05).

Under Cd- and As-contaminated soil, the different phosphorus applications showed significant differences (*p* < 0.05) among all treatments. The maximum hay yield (3566.87 kg·hm^2^) was found in SSP under As- and Cd-contaminated soil and showed significance with the various types of phosphorus fertilizers. The hay yields increased significantly by 69%, 57%, 15%, 48%, 59%, and 0.39% for SSP, DAP, MAP, CaP, HighCaP, and RP, respectively, associated with CK, with the highest yield occurring with SSP application (Figure 2a). The hay yields were in the order of SSP > HighCaP > DAP > CaP > MAP > RP, and the maximum hay yield was found in the SSP treatment.

The root biomass results also showed significant differences among different phosphorus fertilizer applications under Cd- and As-contaminated soil (Figure 2b). The highest root biomass content was found in SSP (1687.093 kg·hm^−2^), followed by CaP, HighCaP, DAP, MAP, and RP. Compared to CK, the root weights of SSP, CaP, HighCaP, DAP, MAP, and RP samples were increased by 97%, 91%, 88%, 56%, 46%, and 3.36%, respectively. The addition of different phosphorus fertilizers enhanced *L. perenne* biomass yields under Cd- and As-contaminated soil, and SSP gave the maximum biomass yield as compared to other treatments.

#### 3.1.3. Root Morphology of *L. perenne*

**Figure 3:**
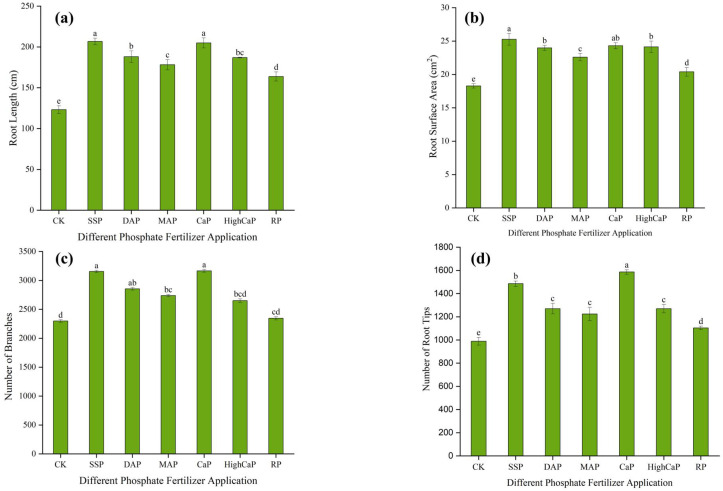
Effects of different phosphorus fertilizer applications on root morphology, including the (**a**) root length, (**b**) root surface area, (**c**) number of branches, and (**d**) number of root tips of *Lolium perenne* under Cd- and As-contaminated soil. Notes: CK: no phosphorus fertilizer application; SSP: single super phosphate; DAP: diammonium phosphate; MAP: monoammonium phosphate; CaP: calcium phosphate; HighCaP: high calcium phosphate; RP: rock phosphate. Different letters correspond to significant differences among different treatments (*p* < 0.05).

The root morphology was affected by the different phosphorus applications under Cd- and As-contaminated soil. Figure 3 shows that the length, surface area, and number of tips of roots in contaminated soil samples treated with phosphorus fertilizer sources were significantly increased (*p* < 0.001).

The total root lengths resulting from different phosphorus fertilizer applications were increased by SSP (67%), DAP (52%), MAP (44%), CaP (66%), HighCaP (52%), and RP (33%), as compared to CK. Plants treated with SSP had greater root lengths (206.5 cm), although the length was not significantly different from CaP (204.97 cm) (Figure 3a). Regarding the root surface area, the greatest surface area (25.28 cm^2^) occurred in SSP, with a 38% larger surface area than CK (Figure 3b). In terms of the number of branches, more branches were observed in SSP (37%) and CaP (37%), followed by DAP (24%), MAP (19%), HighCaP (15%), and RP (2%) (Figure 3c). The differences were found to be statistically significant at *p* < 0.01. In Figure 3d, a higher tip number was found in CaP (60%), followed by SSP (50%). Among all phosphorus fertilizer applications, the RP values in the root system architecture did not appear to differ from CK. Root growth under CK was the lowest, and this was affected by the presence of Cd and As in the soil.

### 3.2. Effects of Different Phosphorus Fertilizer Applications on P, Cd, and As Contents of Lolium perenne

#### 3.2.1. Total P Concentration in *L. perenne*

**Figure 4:**
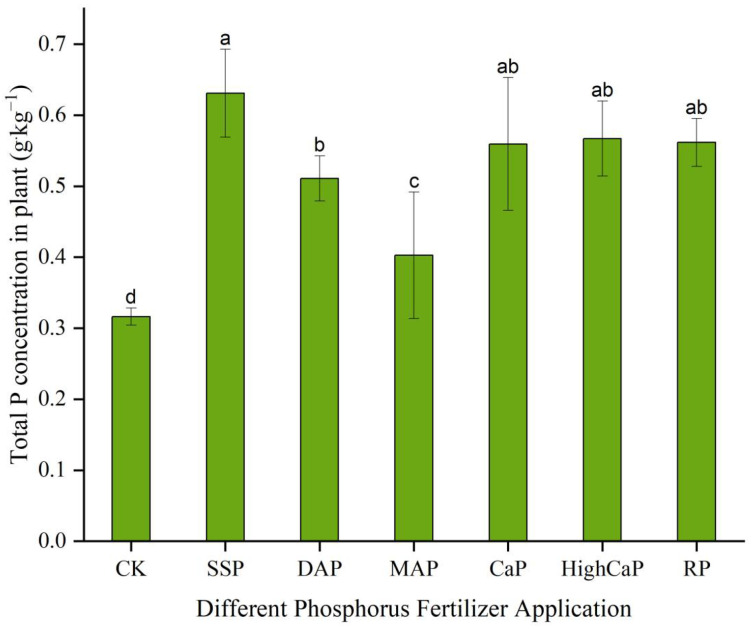
Effects of different phosphorus fertilizer applications on total P content in *Lolium perenne* under Cd- and As-contaminated soil. Notes: CK: no phosphorus fertilizer application; SSP: single super phosphate; DAP: diammonium phosphate; MAP: monoammonium phosphate; CaP: calcium phosphate; HighCaP: high calcium phosphate; RP: rock phosphate. Different letters correspond to significant differences among different treatments (*p* < 0.05).

In soil contaminated with Cd and As, the SSP application enhanced the P content (0.63 g·kg^−1^) in plant shoots, which was significantly (*p* < 0.05) higher than that of other phosphorus fertilizer applications. The highest concentration of P was observed in the SSP treatment, and the lowest was found in CK (Figure 4). The total P concentrations were in the order of SSP > HighCaP > RP > CaP > DAP > MAP > CK. SSP had a higher (102%) P concentration than CK, and HighCaP (82.14%), RP (80.37%), CaP (79.69%), DAP (64.10%), and MAP (29.36%) were also higher.

#### 3.2.2. Cadmium Accumulation in *L. perenne*

**Figure 5:**
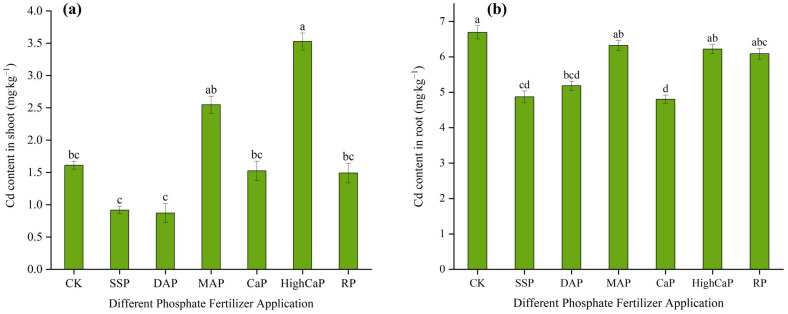
Effects of different phosphorus fertilizer applications on Cd contents in (**a**) shoots and (**b**) roots of *Lolium perenne* under Cd- and As-contaminated soil. Notes: CK: no phosphorus fertilizer application; SSP: single super phosphate; DAP: diammonium phosphate; MAP: monoammonium phosphate; CaP: calcium phosphate; HighCaP: high calcium phosphate; RP: rock phosphate. Different letters correspond to significant differences among different treatments (*p* < 0.05).

Under Cd- and As-contaminated soil, the Cd contents in the shoots were significantly different (*p* < 0.05) among treatments (Figure 5a). Among all treatments, DAP was the lowest, followed by SSP. The Cd contents in the shoots ranged from 0.87 mg·kg^−1^ to 3.5 mg·kg^−1^. The Cd contents in SSP, DAP, CaP, and RP decreased to −43%, −45%, −5.3%, and −7.4% compared to CK. However, the Cd contents of MAP and HighCaP increased by 58% and 118%, respectively, compared to CK. The Cd contents in the roots were also significantly different (*p* < 0.05), with the highest content found in CK (Figure 5b). The Cd content of the CaP treatment was the lowest (−28%), followed by SSP (−27%), DAP (−22%), RP (−9%), HighCaP (−7%), and MAP (−5%), when compared with CK.

#### 3.2.3. As Accumulation in *L. perenne*

**Figure 6:**
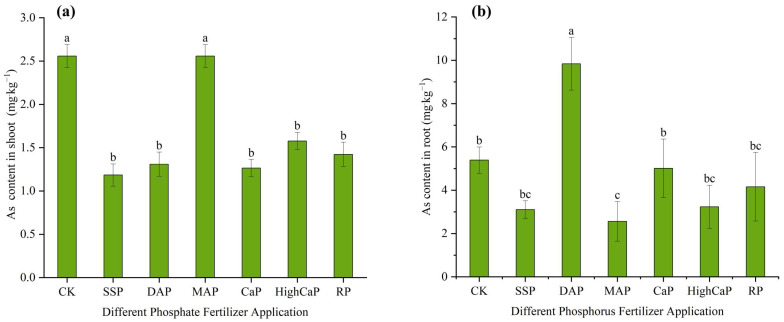
Effects of different phosphorus fertilizer applications on As contents in (**a**) shoots and (**b**) roots of *Lolium perenne* under Cd- and As-contaminated soil. Notes: CK: no phosphorus fertilizer application; SSP: single super phosphate; DAP: diammonium phosphate; MAP: monoammonium phosphate; CaP: calcium phosphate; HighCaP: high calcium phosphate; RP: rock phosphate. Different letters correspond to significant differences among different treatments (*p* < 0.05).

Under Cd- and As-contaminated soil, the As contents in the shoots were significantly different (*p* < 0.05) among all treatments, and the As contents ranged from 1.18 to 2.56 mg·kg^−1^. Among all treatments, MAP was the highest and was not distinct from CK (Figure 6a). When compared with CK, the contents of SSP, DAP, CaP, HighCaP, and RP decreased to −53%, −48%, −50%, −38%, and −44%, in the order of SSP < CaP < DAP < RP < HighCaP. The lowest value was found in SSP, followed by DAP. The As contents in the roots were also highly significantly different (*p* < 0.001); MAP was the lowest (−52%), followed by SSP (−48%), as compared to CK (Figure 6b).

### 3.3. Effects of Different Phosphorus Fertilizer Applications on Antioxidant Enzyme and Non-Antioxidant Enzyme Activities in Plants

#### 3.3.1. Antioxidant Enzyme Activities in *L. perenne*

**Figure 7:**
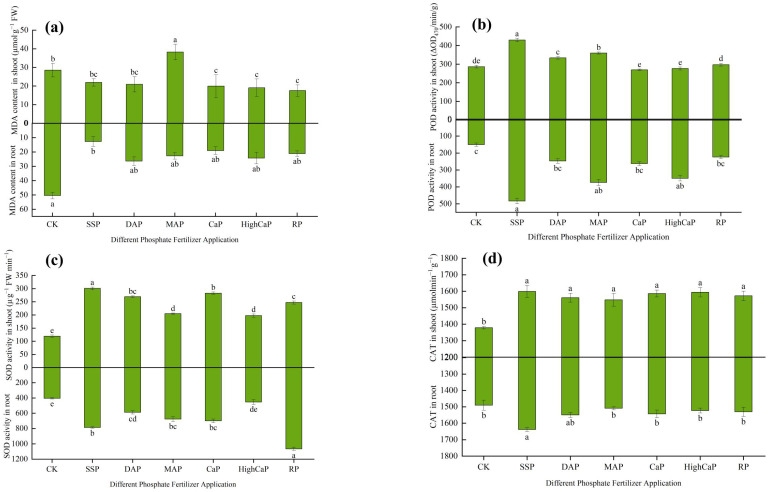
Effects of different phosphorus fertilizer applications on (**a**) MDA contents, (**b**) POD activities, (**c**) SOD activities, and (**d**) CAT activities in *Lolium perenne* under Cd- and As-contaminated soil. Notes: CK: no phosphorus fertilizer application; SSP: single super phosphate; DAP: diammonium phosphate; MAP: monoammonium phosphate; CaP: calcium phosphate; HighCaP: high calcium phosphate; RP: rock phosphate. Different letters correspond to significant differences among different treatments (*p* < 0.05).

Under Cd- and As-contaminated soil, the MDA contents in the shoots were significantly increased (*p* < 0.05). The MDA contents for CK, SSP, DAP, MAP, CaP, HighCaP, and RP were 28.5, 25.3, 20.9, 38.3, 20.0, 19.1, 17.5 µmol·g^−1^ FW, respectively, and the highest content was found in MAP (38.3 µmol·g^−1^ FW), followed by CK (Figure 7a). The MDA content increased by 34% in the MAP treatment and by −23%, −26%, −29%, −33%, −38% decreased in SSP, DAP, CaP, HighCaP, and RP, respectively, over CK. The MDA contents in the roots were not significantly different across the treatments but the content was higher in CK (50.6 µmol·g^−1^ FW) than other phosphorus fertilizer applications. The MDA content increased in CK and decreased by −74%, −47%, −50%, −62%, −51%, −58% in SSP, DAP, MAP, CaP, HighCaP, and RP, respectively, when compared with CK. The lowest MDA content in the roots was observed in SSP (12.7 µmolg^−1^ FW).

Under Cd- and As-contaminated soil, the POD activity levels in the shoots and roots were higher in SSP than the other phosphorus fertilizer applications (Figure 7b). The POD contents in the shoots were significantly different to the control (*p* < 0.001), along with significantly different (*p* < 0.05) POD contents in the roots. The POD activity rates in the shoots after different phosphorus applications were in the order of CK (286), SSP (428), DAP (334), (MAP) 360, CaP (270), HighCaP (276), and RP (297) (ΔOD_470_/min/g). Increases of 49%, 16%, and 25% in POD activity were found in SSP, DAP, and MAP, while decreasing activity levels were found in CaP (−5.4%), HighCaP (−3.3%), and RP (−3.7%) compared with CK. The POD activity rates in the roots were in the order of SSP > MAP > HighCaP > CaP > DAP > RP > CK, with respective increases of 212%, 148%, 132%, 74%, 64%, and 48% over CK. The lowest POD activities in the shoots and roots were found in CK.

Under Cd- and As-contaminated soil, the SOD activities of *L. perenne* shoots were significantly different among the different phosphorus fertilizer application treatments (*p* < 0.001). Higher SOD activity in the shoots occurred in SSP (301.16 µ·g^−1^FWmin^−1^). Under Cd- and As-contaminated soil, the order of SOD activity levels in the shoots was SSP > CaP > DAP > RP > MAP > HighCaP > CK, with an increase of 152% in SSP compared with CK. The SOD activity levels in the roots were significantly different among all treatments (*p* ≤ 0.01), and the greatest SOD activity was found in RP (1060.8 µg^−1^FWmin^−1^), followed by SSP (800.1 µ·g^−1^FWmin^−1^). The trend of SOD activity levels in the roots was found to be in the order of RP > SSP > CaP > MAP > DAP > HighCaP > CK. The lowest SOD activity levels in the roots and shoots were found in CK (Figure 7c).

Under Cd- and As-contaminated soil, the CAT activities in the shoots under different phosphorus fertilizer application treatments were significantly different (*p* ≤ 0.01) compared with CK, and the highest content was found in SSP (Figure 7d). The CAT activities in the shoots of CK, SSP, DAP, MAP, CaP, HighCaP, and RP were 1378.689, 1598.59, 1560.67, 1547.6, 1586.16, 1593.68, and 1572.14 µmolmin^−1^·g^−1^, respectively. The CAT activities in the SSP, CaP, and HighCaP treatments were the same (15%), while increases of 13%, 12%, and 14% were found in DAP, MAP, and RP over CK, respectively. However, the CAT activities in the roots were not significantly different from each other at 1490.404, 1638.584, 1549.676, 1509.046, 1543.462, 1522.908, and 1530.556 µmolmin^−1^·g^−1^, respectively. The CAT activities occurred in sequence of SSP > DAP > CaP > RP > HighCaP > MAP > CK, with increases of 9.9%, 3.9%, 3.5%, 2.6%, 2.1%, and 1.2% over CK, respectively.

#### 3.3.2. Non-Antioxidant Enzyme Activities in *L. perenne*

**Figure 8:**
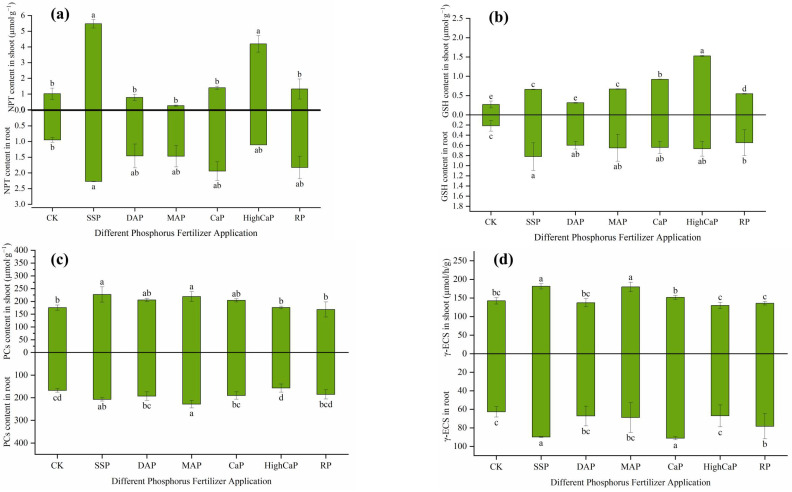
Effects of different phosphorus fertilizer applications on (**a**) NPT contents, (**b**) GSH contents, (**c**) PCs contents, and (**d**) γ-ECS contents of *Lolium perenne* under Cd- and As-contaminated soil. Notes: CK: no phosphorus fertilizer application; SSP: single super phosphate; DAP: diammonium phosphate; MAP: monoammonium phosphate; CaP: calcium phosphate; HighCaP: high calcium phosphate; RP: rock phosphate. Different letters correspond to significant differences among different treatments (*p* < 0.05).

Under Cd- and As-contaminated soil with different phosphorus fertilizer applications, the NPT contents in ryegrass shoots of CK, SSP, DAP, MAP, CaP, HighCaP, and RP were 1.0162, 5.47785, 0.78251, 0.258972, 1.39609, 4.1959, and 1.31955 µmol·g^−1^, respectively. The NPT contents in the roots were 0.948, 2.2705, 1.457, 1.468, 1.9405, 1.108, 1.8285, and 1.31955 µmol·g^−1^, respectively. Different phosphorus fertilizer applications showed significantly higher values than the control (*p* < 0.05) in terms of the NPT content of the shoots, but they were not significantly different in the roots. The highest NPT contents in the shoots and roots were found in the SSP treatment (Figure 8a). The lowest NPT content in the shoots was found in MAP, while CK had the lowest content in the roots.

Under Cd- and As-contaminated soil, the different phosphorus fertilizer applications showed highly significant differences in the GSH contents of the shoots and roots (*p* < 0.05) (Figure 8b). The GSH contents in the shoots of CK, SSP, DAP, MAP, CaP, HighCaP, and RP were 0.27, 0.67, 0.32, 0.67, 1.15, 2.38, and 0.5415 µmol·g^−1^, respectively, and the GSH contents in the roots were 0.216, 0.822, 0.5987, 0.6507, 0.642, 0.6677, and 0.5487 µmol·g^−1^, respectively. The GSH contents in the shoots were observed in the following order: CK < DAP < RP < MAP < SSP < CaP < HighCaP. The highest GSH content was found in HighCaP (758.633%) as compared to CK. The highest GSH content in the roots was observed in SSP (280.55%), while decreasing GSH contents were found in the order of CK < RP < DAP < CaP < MAP < HighCaP < SSP.

The PCs contents were significantly different in both the shoots and roots of *L. perenne* (Figure 8c). The shoots’ PCs activities for different phosphorus fertilizer applications under Cd- and As-contaminated soil were 175.71, 224.06, 205.82, 219.06, 204.06, 176.05, and 168.43 µmol·g^−1^, respectively, while values of 167.66, 207.37, 192.84, 228.11, 190.11, 157.08, and 185.14 µmol·g^−1^, respectively, were found in the roots. In the shoots, except for RP (−4.14%), the SSP, DAP, MAP, CaP, and HighCaP treatments showed increases of 27.5%, 17.1%, 24.6%, 16.1%, and 0.19% compared to CK. In terms of the roots, except for HighCaP (−6.31%), all treatments were increased compared to CK. MAP had the highest PCs content, which increased by 36.1% over CK.

Under Cd- and As-contaminated soil, the different phosphorus fertilizer applications showed significant differences in the shoots (*p <* 0.05) but not in the roots (Figure 8d). Low γ-ECS activities in both the shoots and roots were observed in CK. The γ-ECS activities in the shoots showed decreases in DAP (−3.55%), RP (−4.58%), and HighCaP (−8.61%) but increases in CaP (6.53%), MAP (26.44%), and SSP (27.52%) compared to CK. In the roots, the lowest γ-ECS activity (6.85%) was found in the HighCaP treatment and the highest activity was found in SSP among all phosphorus fertilizer treatments.

### 3.4. Soil Rhizosphere Microbial Community

The analysis revealed that the different phosphorus fertilizer applications were the main factor influencing the alpha diversity of bacterial communities based on Chao, Simpson, Shannon, and Pielou diversity indices. The alpha diversity (Chao 1 index, Simpson index, Shannon index, and Pielou index) under different phosphorus fertilizer applications had no significant effect on the alpha diversity indices (*p* > 0.05) (Figure S1a–d).

The analyses of bacterial communities using 16SrRNA sequencing included a principal component analysis (PCA) (Figure S2a) and principal coordinates analysis (PCoA) (R2 = 30.89%, *p >* 0.05, Figure S2b) of data from the beta diversity of soil microbial communities and a permutational multivariate analysis of variance (PerMANOVA), which demonstrated that there was clustering of microbial communities and no significant differences among different phosphorus fertilizer treatments under Cd- and As-contaminated soil.

The beta diversity analysis showed differences in the structures of the bacterial communities in the soil (Figure S2c). Among all treatments, MAP bacterial communities were more variable, indicating higher dissimilarity of the bacteria.

**Figure 9:**
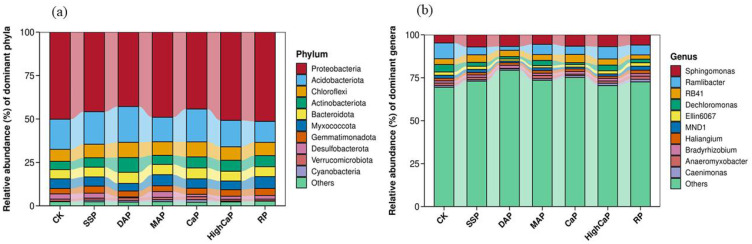
Relative abundance of the most abundant bacterial (**a**) phyla and (**b**) genera after the different phosphorus fertilizer applications under Cd- and As-contaminated soil. Notes: CK: no phosphorus fertilizer application; SSP: single super phosphate; DAP: diammonium phosphate; MAP: monoammonium phosphate; CaP: calcium phosphate; HighCaP: high calcium phosphate; RP: rock phosphate.

The soil bacterial microbiomes we observed mostly accounted for 4.7–50% at the phyla level and 2.27–7.08% at the genera level of the total relative abundance, depending on the different phosphorus applications (Figure 9a). The abundance analysis revealed that Proteobacteria accounted for 40–50% of the communities throughout the six groups of fertilizer-treated soils, indicating that Proteobacteria was the dominant phylum in the soil. The major compositions of bacterial microbiomes after different phosphorus applications under Cd- and As-contaminated soil were mostly of Proteobacteria, Acidobacterioa, Chloroflexi, and Actinobacterioa. The top two dominant bacterial phyla were Proteobacteria and Acidobacteria. The Proteobacteria communities were not different from each other, and the relative abundance levels of Acidobacteria were found to be higher in DAP (20.58%), CaP (18.89%), and SSP (18.72%). At the genus level, the top three identified bacterial genera were *Sphingomonas, Ramlibacter,* and *RB41* (Figure 9b). The relative abundance of *Spingomonas* was higher in all phosphorus fertilizer applications than CK, and 7.08% was found in SSP. In addition, the relative abundance of RB41 was higher in SSP and CaP treatments compared to other treatments.

### 3.5. Correlation Analysis

**Figure 10:**
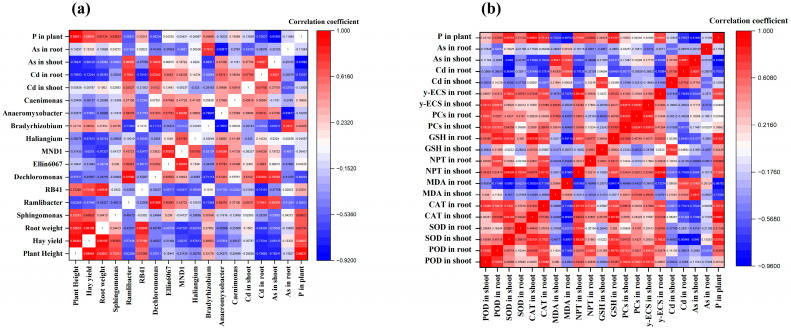
Correlation analysis between (**a**) plant growth characteristics, microbial diversity, and (**b**) physiological characteristics of *Lolium perenne* under Cd- and As-contaminated soil.

According to the correlation analysis, the plants’ phosphorus concentrations and cadmium and arsenic contents were substantially linked with *L. perenne* growth, yield, root biomass, and soil bacterial community results. The correlation analysis was conducted on the changes in plant height, hay yield, plant phosphorus concentration, and other indicators (Figure 10a). It was found that the plant phosphorus concentration was significantly positively correlated with plant height, biomass, Spingomonas, and RB4I at the genus level, and each indicator was also significantly positively correlated with each other. However, it was significantly negatively correlated with the Cd content in the roots and the As content in the shoots of *L. perenne*.

The correlation analysis was conducted on the changes in POD, SOD, CAT, MDA, NPT, GSH, PCs, γ-ECS, plant phosphorus concentration, and Cd and As contents in the shoots and roots of *Lolium perenne* (Figure 10b). It was found that the plant phosphorus concentration was significantly positively correlated with POD, SOD, CAT activity, GSH, PCs content, and γ-ECS levels in the roots, while it was significantly negatively correlated with MDA and Cd and As contents in the shoots and roots of the plants. The Cd and As contents in the shoot and root parts of the plants were significantly negatively correlated with the activities of POD, SOD, and CAT and the contents of NPT, GSH, PCs, and γ-ECS. However, the content of MDA was significantly positively correlated with Cd content in the plants and As content in the shoots of *L. perenne.*

## 4. Discussion

### 4.1. Effects of Different Phosphorus Fertilizer Applications on Growth, Accumulation, and Rhizosphere Microbial Community of Lolium perenne Under Cd- and As-Contaminated Soil

Heavy metal contamination in soil, particularly that of Cd and As, reduces plant growth [55]. Furthermore, nutrient absorption and synthesis are closely related to plant biomass and crop yields. As, a recognized toxicant, has both beneficial and detrimental impacts on plant nutritional uptake and accumulation. The use of phosphorus fertilizer has been shown to increase crop development and yields, even when contaminated with Cd and As. In heavy metal-contaminated soil, heavy metals impede plant growth, specifically due to Cd and As pollutants in the soil [55]. Plant biomass and grain productivity have a direct relationship with the uptake of nutrients and assimilation [56]. Our study showed that various types of phosphorus fertilizers enhanced plant growth, with SSP resulting in greater plant height compared to other phosphorus fertilizers. Initially, the plant height increased, but growth did not continue afterwards. Additionally, the biomass, including both root and shoot weights, was also higher in the SSP treatment. Similarly, it was noted by Khan et al. [57] that of the various phosphatic fertilizers, SSP produced the maximum wheat grain yield after nitrophenols and DAP. This suggests that Bambara groundnuts react to varying levels of SSP supplements and that their vegetative development is increased by SSP treatment at different levels. The addition of inorganic P significantly affects Bambara groundnut growth and production [58]. Mani et al. [59] found that 200 kg ha^−1^ of SSP increased the dry biomass of carrots to 498 g plot^−1^, which was 26.50% more than the control and amply demonstrated the beneficial effects of SSP on carrot dry biomass output.

Enhancing fertilizer application methods requires elucidating how P buildup affects soybean dry matter yields [60]. P encourages an increase in plant biomass and is essential for several metabolic activities [61]. The most popular and established approach to increasing and precipitating the constant P in cropping soil for the crops to efficiently absorb is chemical P fertilizer application in croplands [62]. In this study, the P concentration in Cd- and As-contaminated soil was higher in SSP, followed by HighCaP, RP, and CaP. However, DAP and MAP had lower P concentrations. Zhou et al. [63] showed that SSP and CMP treatments outperformed MAP and DAP in terms of yield and P accumulation, resulting in a greater P fertilizer impact and increased P uptake in plants for CMP and SSP. According to the correlation study, a positive relationship was found between plant development, yield, and P concentration. Additionally, the content of SSP in plants was greater compared to that of other P fertilizer resources, particularly DAP and MAP. Depending on how well it dissolves and the types that plants can absorb, it may also be discovered that P supplies increase biomass and P concentrations of all parts through all stages of development [64]. According to the preceding research, better yields were obtained when low-P fertilizer sources, such as SSP, were applied to greater plant populations as opposed to higher-P fertilizer sources, such as MAP or DAP [65]. When P fertilizers containing high concentrations are applied to soil, their absorption leads to a rapid decrease in water solubility, which makes them less bioavailable [66].

When the roots absorb Cd, this causes several changes in the morphological characteristics and physiochemical functions of the plant. The main way that plants absorb Cd is from their roots [67], and as a result, these metal ions hurt them [68]. Fertilized plants responded significantly to Cd and As stress in comparison with CK. These root morphology metrics were significantly impacted by the types of fertilizer and the amounts of phosphorus applied. Various types of phosphorus fertilizer administration increased the root biomass and morphology, whereas the control (CK) showed an impaired root structure. Better performance of the root system (biomass, length, surface area, and branches) was found in SSP (Figure 2b and Figure 3a–c). P fertilizers encourage root development by increasing the root surface area and length, which improves the ability of plants to absorb soil nutrients. This may be because the combined pollution of Cd and As decreases root development and lengthening [69]. Shanying et al. [70] reported that the harmfulness of Cd and As is typically marked by a decrease in root length, changes to the root system, and a decline in the creation of root hairs. Furthermore, the P supplementation under Cd stress reduced the Cd concentrations in different plant parts by improving the root growth and formation [71].

The contents of Cd and As in both the roots and shoots of *L. perenne* were also lower in most phosphate fertilizer treatments and especially lower in SSP than in other phosphate fertilizer application treatments. It was found that the P concentration had a negative relationship with Cd and As contents in plants and a positive correlation with the plant growth and yield of *L. perenne*. These results were due to the interactions between phosphate and Cd, as well as phosphate and As, in the soil–plant relationship. P often lowers the absorption of As (mostly arsenate) in plant and microbes, impacting As transport [72]. Additionally, Wang et al. [73] verified that the usage of phosphate in contaminated soils caused a considerable decline in the concentration of Cd in plants, demonstrating the apparent antagonistic influence of phosphorous (P) on the mobility of Cd. Nevertheless, precipitating activities, including phosphates and carbonates, dominate cadmium migration in alkaline soils [74]. Using a variety of processes, especially precipitation, sorption, or co-precipitation, the presence of SSP may cause decreased Cd phytoavailability [75]. Cd application at 15 mg kg^−1^ + SSP 200 kg ha^−1^ also reduced the uptake of Cd to 0.88 mg kg^−1^ in the carrot’s shoots and 1.08 mg kg^−1^ in the roots [58]. Furthermore, P-Cd interactions produce Cd-P compounds, or Cd_3_(PO_4_)^2^, which may reduce Cd toxicity [76], reduce Cd in crops [77], and encourage plant development [78].

The function of microorganisms is to either increase plants’ resistance to heavy metals or decrease their uptake of cadmium [79]. Another investigation into soil microorganisms suggested that cadmium and other heavy metals can impact microbial populations, particularly bacteria, by altering the enzyme activity, denaturalizing proteins, or destroying the cellular structure (lipid or protein bonding structures). These effects can then have an impact on the microbial population and its interaction with plants [80]. At the phylum level, Proteobacteria and Acidobacteria were found as the dominant groups, showing important roles in nutrient cycling and metal elimination. Similarly, according to earlier research on cold-seep sediments, Proteobacteria are key phyla in the elemental processes of sulfur, nitrogen, and carbon [81]. The majority of the main microorganisms, which included all Gram-negative Proteobacteria, were involved in the nitrogen cycle, a crucial mechanism in the soil microbial community. Each of these bacteria played a part in the microorganisms’ ability to tolerate harmful metals [82]. Proteobacteria were consistently observed to be likely to retain or expand their dominance under Cd stress among the major bacterial lineages [83]. This is likely because the phylum contains a variety of Cd-resistant strains. The rhizosphere microenvironment can be improved by beneficial microbial consortia, which may lessen the damaging influences of heavy metal impacts on plants [84]. The growth and development of native plants, in addition to the structure and ecological role in the bacterial groups related to them, are known to be adversely impacted by Cd contamination in soils [85]. Furthermore, due to their positive effects on promoting growth of plants in contaminated soils with metals and their possible role in mitigating HM-induced toxicity, plant–microbe interactions in soil have enhanced phytoextraction efficiencies [86]. In this study, we observed that Sphingomonas bacteria were dominant and were higher in the SSP application. Notably, the dominant bacteria, such as Spingomonas and RB41, show significant positive associations with plant growth, biomass, and P concentration in plants, as well as a significantly negative correlation with the contents of Cd and As seen in shoots and roots (Figure 10a). This was found in rhizosphere soil, where bacterial species such as Spingomonas were dominant, promoting plant growth well and also reducing metal stress under Cd- and As-contaminated soil. This was confirmed when the presence of Sphingomonas at the genus level proved more effective in mitigating metal contamination, and its resilience to different toxic metals suggests that it might be used as a bioreactor for eliminating heavy metal contamination [87]. Additionally, the activity and quantity of Sphingomonas in the rhizosphere, endosphere, or phyllosphere promote the mineralization, degradation, stabilization, or confinement of refractory pollutants [88].

### 4.2. Physiological Mechanisms of the Effects of Different Phosphorus Fertilizer Applications on Lolium perenne Under Cd- and As-Contaminated Soil

Plants produce many reactive oxygen species (ROS) due to the occurrence of Cd and As stress, which damages the cell membrane’s composition. Antioxidant substances, whether endogenous or exogenous, can eliminate ROS and lessen cellular molecule oxidation processes, thereby reducing the harm caused by an excess of ROS [89], but MDA, a cytotoxic byproduct of lipid peroxidation, is a sign of the formation of free radicals and the resulting tissue damage [90]. Accordingly, the amount of ROS and the extent of plasma membrane destruction in plants can be verified by assessing the quantity of MDA [79]. An essential defensive mechanism for eliminating reactive oxygen species is the antioxidant response [91]. Enzymatic antioxidants are typically altered in response to cadmium exposure. An adaptive reaction to the harm caused by H_2_O_2_, which is created during cell metabolism, is the rise in CAT activity [92]. The results showed that different phosphate fertilizer applications increased the activities of POD, SOD, and CAT in both shoots and roots. This increase might be because of Cd stress [93]. P treatment in wheat (*Triticum aestivum*) decreased Cd^2+^ uptake while simultaneously enhancing the action of antioxidant enzymes, such as SOD, POD, and CAT. Additionally, Maqbool et al. [94] discovered that superoxide dismutase, ascorbate peroxidase, peroxidase, catalase, and enzyme activity levels were significantly enhanced after applying P fertilizer as compared to the control. The findings demonstrated that MAP application led to higher MDA concentrations in the shoots, suggesting that plants treated with MAP may be less resilient to stress than plants treated with other phosphate fertilizer supplies. The highest SOD activity in the shoots and POD and CAT activities in both the shoots and roots were observed in SSP among all other treatments, but the MDA content was decreased in SSP. Maqbool et al. [94] also discovered that MDA activity decreased with P fertilizer treatment. Electrolyte leakage in foliage after SSP, DAP, and combined DAP and SSP treatments was significantly reduced by 15, 9, and 43%, respectively.

In reaction to Cd and As pollution in soil, HMs ions were chelated by non-antioxidant compounds and their associated enzymes (NPT, GSH, GSS, PCs, γ-ECS, AR, and PCSase) [95]. GSH and PCs are the main compounds associated with the plant’s responses to As and Cd, although there was some resemblance between the toxicity and chelate process of these two metals in cells [96]. This study indicated that except for DAP, all phosphorus-fertilizer-amended treatments yielded higher GSH contents. A lower GSH content was found in the shoots of SSP plants, but the GSH content in the roots was higher in SSP-treated plants. Since GSH may indirectly function as a predecessor of PCs, a ligand peptide with an exceptionally high attraction for certain heavy metals, low GSH levels can result in a rise in PCs [97]. Higher NPT and PCs contents were found in SSP. The SSP application resulted in higher PCs and lower Cd and As contents in plants, showing a negative correlation between PCs and Cd and As contents in plant parts. The results showed that plants treated with SSP fertilizer can be resistant to Cd and As stress, with a better oxidative stress defense system, because in plants the top-heavy metal chelators are phytochelatins (PCs), particularly when it comes to resistance to Cd [98]. Additionally, plants generate PC–metal complexes to sequester metals. Another crucial function of PCs is the transmission of Cd from roots to shoots [99].

Through thiol-based oxidative stimulation or feedback suppression by GSH, GSH levels can be controlled under stress circumstances at a certain level of γ-ECS activity [100]. The results stated that γ-ECS activities in the SSP treatment samples increased in the roots and shoots of *L. perenne*, also supporting reduced metal transport in the plant. To detoxify As and Cd, more GSH and PCs must create Cd^2+^/As^3+^-PC or -GS2/GS3 interactions [101]. The co-occurrence of γ-ECS influenced the contents of Cd and As in perennial ryegrass, enhancing the synthesis of plant-chelating enzymes. This activity resulted in a greater elimination of ROS related to Cd and As, thereby decreasing the harmful effects of ryegrass under mixed contamination [50].

Overall, the SSP application exhibited high SOD, POD, and CAT activities, while we observed low levels of MDA. According to Li et al. [102], the reduced MDA amount may lower the quantity of ROS and Cd bioavailability. The activities of SOD, POD, CAT, and NPT in the shoots; GSH in the roots; and PCs, γ-ECS contents in the shoot and root were negatively correlated with Cd contents in the plants. Regardless, MDA contents were positively associated with Cd contents in plants and As content in shoots (Figure 10b). Under Cd and As stress, the antioxidant enzyme activities, including SOD, CAT, and POD, are typically altered. Additionally, the antioxidant enzyme complex is where plants detoxify. POD and CAT scavenge plant tissues for H_2_O_2_ and catalyze the breakdown of H_2_O_2_ into H_2_O, whereas SOD can scavenge O^2−^ in plant cells to create harmless O_2_ with minimal toxicity H_2_O_2_ [102]. A low MDA content was positively correlated with Cd and As contents in plants, indicating low oxidative stress damage. This correlation was related to lipid peroxidation of the plant membrane, which deepened with the phosphorus fertilizer sources, thereby enhancing responses to Cd and As damage. Liu et al. [103] described that PCs primarily serve two purposes—purifying plant cells and enhancing plant defenses against pollutants and heavy metal chelation. In particular, the application of SSP improves the PCs content in plants and can deactivate the bioavailability of Cd and As to plants, as the primary function of PCs is to bind metal ions through metal complexation. Furthermore, one method of increasing plants’ ability to resist heavy metals is through PC–metal complexes. PCs is currently the most important of the metal chelators that function to mitigate the harmful effects of specific dangerous HMs, demonstrating that PCs’s functions include shielding plants from harmful metals. Under stress conditions, GSH levels can be regulated by the γ-ECS function via thiol-created oxidative stimulation or through reaction–restriction by GSH [100].

Accordingly, the findings showed that the application of phosphorus fertilizer sources significantly improved the yield of *L. perenne* by reducing Cd and As accumulation in plants. Among the different sources, SSP proved most effective, simultaneously lowering Cd and As uptake, enhancing antioxidant defense responses, and fostering a healthier rhizosphere microbial community.

## 5. Conclusions

In the present study, the application of SSP was efficacious in enhancing hay yields (3567.6 kg·hm^−2^) and also increasing antioxidant enzyme activities, including POD, SOD, NPT, and GSH activities in the roots and PCs and γ-ECS contents, while lowering MDA contents compared with other phosphorus fertilizer sources. When compared to CK, the SSP application decreased the Cd contents by −43% in the shoots and −27% in the roots, as well as the As contents by −53% in the shoots and −48% in the roots. Greater accumulation of P and reduced uptake of Cd and As in plants were observed with the application of SSP, indicating its ameliorating effect on Cd- and As-contaminated soil. The findings also revealed that SSP was efficiently higher at 7.08% in terms of *Spingomonas* abundance, which may impede Cd uptake by activating *Spingomonas* in the soil rhizosphere. Therefore, SSP shows strong potential to alleviate metal stress in the soil, possibly reducing Cd and As in forage and helping to mitigate the risk of toxicity in perennial ryegrass production in Cd- and As-contaminated conditions. However, the present investigation was conducted under pot-based greenhouse conditions. Consequently, additional field studies are necessary to validate the findings and to clarify the physiological mechanisms and microbial community interactions involved in mitigating Cd and As toxicity, thereby supporting improved forage production in metal-contaminated soils.

## Figures and Tables

**Table 1:** Available Cd and As contents from different sources of phosphorus fertilizer.

Phosphorus Fertilizer Sources	Available Cd Content (mg·kg^−1^)	Available As Content (mg·kg^−1^)
Single super phosphate	0.742	0.366
Diammonium phosphate	4.439	8.732
Monoammonium phosphate	2.833	0
Calcium phosphate	6.226	8.606
High Calcium phosphate	6.348	9.208
Rock phosphate	0	0

## Data Availability

The original contribution for this study is included in the article.

## Supplementary Materials

The following supporting information can be downloaded at https://www.mdpi.com/article/10.3390/toxics13090805/s1: Figure S1. Comparison of alpha diversity: (a) Chao1 index; (b) Shannon index; (c) Simpson index; (d) Pielou index. Alpha diversity of rhizosphere microbial communities after the different phosphate fertilizer applications in Cd- and As-contaminated soil. Figure S2. (a) PCA plot explaining 58.98% (PC1) and 8.19% (PC2) of the variation in microbial communities. (b) PCoA plot explaining 27% (PC1) and 12% (PC2) of the variation in microbial communities. (c) Beta diversity analysis.

## Abbreviations

The following abbreviations are used in this manuscript:

SSP	Single Super Phosphate
DAP	Diammonium Phosphate
MAP	Monoammonium Phosphate
CaP	Calcium Phosphate
HighCaP	High Calcium Phosphate
RP	Rock Phosphate
Cd	Cadmium
As	Arsenic
POD	Peroxidase
SOD	Superoxide dismutase
CAT	Catalase
MDA	Malondialdehyde
NPT	Non-Protein Sulfhydryl
GSH	Glutathione
PCs	Phytochelatin Synthetase
γ-ECS	γ-Glutamylcysteine Synthetase
