# Supplementary material for: Single Super Phosphate Improves Lolium perenne Quality and Rhizosphere Microorganism Structure Under Combined Cadmium and Arsenic Stress
Source: Toxics. 2025 Sep 22;13(9):805. doi: 10.3390/toxics13090805 (PMC12474417; doi:10.3390/toxics13090805)
Supplement: Supplementary file 1 [file toxics-13-00805-s001.zip › toxics-3844884-supplementary.pdf]

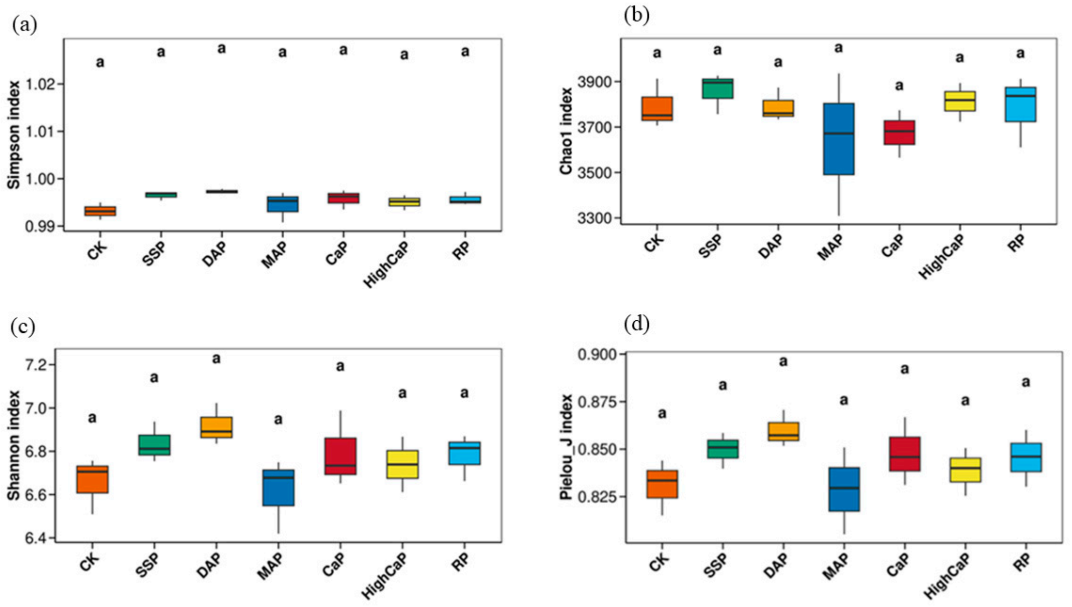

Figure S1 Comparison of alpha diversity (a) Chao1 index, (b) Shannon index, (c) Simpson index and (d) Pielou index; Alpha diversity of rhizosphere microbial communities among the different phosphorus fertilizer application in Cd and As contaminated soil.

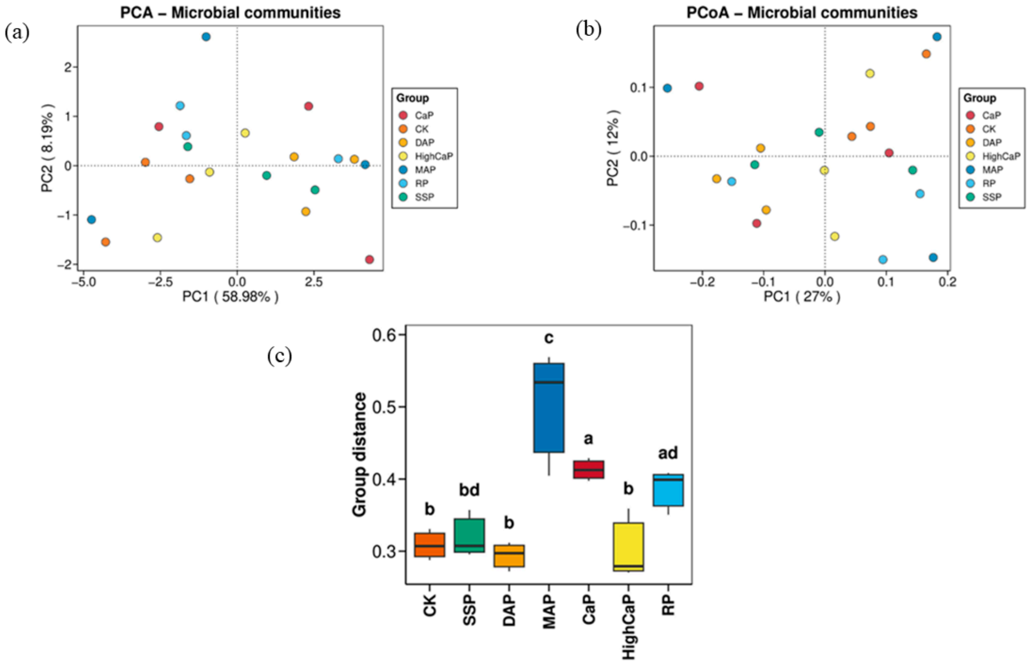

Figure S2 (a) PCA plot explained 58.98% (PC1) and 8.19% (PC2) of the variation in microbial communities, respectively, (b) PCoA plot explained 27% (PC1) and 12% (PC2) of the variation in microbial communities, respectively and (c) Beta diversity analysis.
